# Engineered Bacteriophages Containing Anti-CRISPR Suppress Infection of Antibiotic-Resistant P. aeruginosa

**DOI:** 10.1128/spectrum.01602-22

**Published:** 2022-08-16

**Authors:** Shugang Qin, Yongan Liu, Yuting Chen, Jinrong Hu, Wen Xiao, Xiaoshan Tang, Guohong Li, Ping Lin, Qinqin Pu, Qun Wu, Chuanmin Zhou, Biao Wang, Pan Gao, Zhihan Wang, Aixin Yan, Khan Nadeem, Zhenwei Xia, Min Wu

**Affiliations:** a Department of Critical Care Medicine, Frontiers Science Center for Disease-related Molecular Network, State Key Laboratory of Biotherapy and Cancer Center, West China Hospital, Sichuan University, Chengdu, China; b Department of Biomedical Sciences, School of Medicine and Health Sciences, University of North Dakota, Grand Forks, North Dakota, USA; c Department of Critical Care Medicine, Ruijin Hospital, Shanghai Jiao Tong University School of Medicine, Shanghai, China; d West China Fourth Hospital, Sichuan University, Chengdu, Sichuan, China; e School of Biological Sciences, The University of Hong Kong, Shatin, Hong Kong SAR; f Department of Pediatrics, Ruijin Hospital affiliated with Shanghai Jiao Tong University School of Medicine, Shanghai, China; University of California, San Diego

**Keywords:** *P. aeruginosa*, bacteriophages, CRISPR-Cas, Acr, multidrug resistance bacterial infection, anti-CRISPR

## Abstract

The therapeutic use of bacteriophages (phages) provides great promise for treating multidrug-resistant (MDR) bacterial infections. However, an incomplete understanding of the interactions between phages and bacteria has negatively impacted the application of phage therapy. Here, we explored engineered anti-CRISPR (Acr) gene-containing phages (EATPs, eat Pseudomonas) by introducing Type I anti-CRISPR (*AcrIF1*, *AcrIF2*, and *AcrIF3*) genes into the P. aeruginosa bacteriophage DMS3/DMS3m to render the potential for blocking P. aeruginosa replication and infection. In order to achieve effective antibacterial activities along with high safety against clinically isolated MDR P. aeruginosa through an anti-CRISPR immunity mechanism *in vitro* and *in vivo*, the inhibitory concentration for EATPs was 1 × 10^8^ PFU/mL with a multiplicity of infection value of 0.2. In addition, the EATPs significantly suppressed the antibiotic resistance caused by a highly antibiotic-resistant PA14 infection. Collectively, these findings provide evidence that engineered phages may be an alternative, viable approach by which to treat patients with an intractable bacterial infection, especially an infection by clinically MDR bacteria that are unresponsive to conventional antibiotic therapy.

**IMPORTANCE** Pseudomonas aeruginosa (P. aeruginosa) is an opportunistic Gram-negative bacterium that causes severe infection in immune-weakened individuals, especially patients with cystic fibrosis, burn wounds, cancer, or chronic obstructive pulmonary disease (COPD). Treating P. aeruginosa infection with conventional antibiotics is difficult due to its intrinsic multidrug resistance. Engineered bacteriophage therapeutics, acting as highly viable alternative treatments of multidrug-resistant (MDR) bacterial infections, have great potential to break through the evolutionary constraints of bacteriophages to create next-generation antimicrobials. Here, we found that engineered anti-CRISPR (Acr) gene-containing phages (EATPs, eat Pseudomonas) display effective antibacterial activities along with high safety against clinically isolated MDR P. aeruginosa through an anti-CRISPR immunity mechanism *in vitro* and *in vivo*. EATPs also significantly suppressed the antibiotic resistance caused by a highly antibiotic-resistant PA14 infection, which may provide novel insight toward developing bacteriophages to treat patients with intractable bacterial infections, especially infections by clinically MDR bacteria that are unresponsive to conventional antibiotic therapy.

## INTRODUCTION

Antimicrobial resistance is increasingly becoming a major public health concern in the world (1). Epidemiological studies have shown that nearly 700,000 people die of antibiotic-resistant bacterial infection each year (2). Multidrug-resistant (MDR) bacteria are not sensitive to conventional antibiotics and require novel therapeutics or alternative drugs (3). Pseudomonas aeruginosa (P. aeruginosa) is an opportunistic Gram-negative bacterium that causes severe infection in immune-weakened individuals, especially patients with cystic fibrosis, burn wounds, cancer, chronic obstructive pulmonary disease (COPD), and ventilator-associated pneumonia derived from various severe respiratory diseases, including COVID-19 (4). Once colonized, P. aeruginosa is extremely difficult to eliminate due to its intrinsic multidrug resistance (5). Prevention Health Care-Related Epidemiological Survey Infections reported that almost 9% of infections were caused by P. aeruginosa, the fourth most common pathogen in European hospitals (6). Hospital-acquired infection caused by MDR P. aeruginosa has become a main health threat (2). Hence, novel or alternative therapy is urgently needed to counteract this bacterium.

Bacteria may be eradicated by the corresponding lytic phages in a process known as phage therapy, which does not cause drug resistance (7). A series of trials have demonstrated that patients receiving phage therapy exhibited significantly improved immunity with fewer side effects (8, 9). Phage therapy is especially valuable in treating cases that had exhausted all antibiotics and drugs with MDR Acinetobacter baumannii (10). This attests that the therapeutic use of phages would be a highly viable alternative treatment of MDR bacterial infections (11). However, phage therapy has received limited clinical attention for MDR bacterial infection due to its intrinsic limitations (such as phage tolerance, the restricted host ranges of phages, symbiosis but not lysis, and others) and insufficient knowledge of the arms race between phages and bacteria. Recently, mutations in phage host range-determining regions (HRDRs) that cope with the resistance mutation of bacteria have been implemented as an effective method to prevent Escherichia coli infection (12). Engineered bacteriophages BPsΔ33HTH_HRM10 and D29_HRMGD40 in a cocktail cured a cystic fibrosis patient with a disseminated drug-resistant Mycobacterium abscessus, thereby rendering the rationale for the development of engineered phages and the design of therapeutics to break through the evolutionary constraints of bacteriophages and create next-generation antimicrobials (13, 14).

Clustered regularly interspaced short palindromic repeats (CRISPR) and associated (Cas) proteins act as one of the most widely regarded defense systems against foreign genetic elements in certain bacteria (~40%) and most archaea (~90%) (15). Consequently, various corresponding anti-CRISPR (Acr) proteins have evolved by phages to inhibit bacterial CRISPR systems (16–23). Our previous research identified a series of Type VI-A anti-CRISPR (acrVIA1-7) genes that block the activities of the Type VI-A CRISPR-Cas13a system (23) and also designed Type III CRISPR endonuclease antivirals for coronaviruses (TEAR-CoV) as an experimental therapeutic to combat SARS-CoV-2 infection (24). These studies suggest that Acrs and CRISPR endonucleases possess great potential for the treatment of bacterial or other microorganism infections. Type I-F CRISPR-Cas systems are prevalent in clinically isolated P. aeruginosa (25). Type I-F anti-CRISPR proteins AcrIF1, AcrIF2, and AcrIF3, as potent Type I-F CRISPR-Cas inhibitors, were the earliest to show suppression of Type I-F CRISPR-Cas systems, thereby promoting phage invasion and expansion in bacteria. Importantly, their inhibition mechanisms have been elaborated; AcrIF1 prevents hybridization between target DNA and crRNA by binding to the Cas7f backbone of the Csy complex, AcrIF2 blocks PAM binding sites on double-stranded DNA, and AcrIF3 inhibits Type I CRISPR-Cas immunity by directly binding to Cas3 (26, 27). Overall, these results suggest that AcrIF1-3 have promising for treating P. aeruginosa infections, especially those of clinically isolated MDR strains.

Here, we created engineered anti-CRISPR (*AcrIF1*, *AcrIF2*, and *AcrIF3*) gene-containing phages (EATPs, eat Pseudomonas), tested their potential effects on overcoming CRISPR-Cas bacterial immunity, and analyzed their therapeutic effects on infections of clinically MDR pathogenic P. aeruginosa in human embryonic kidney 293 (HEK293T) cells and mouse respiratory infection models. Our data showed that successful phage replication is correlated with the competition between CRISPR and Acrs. The absolute inhibitory concentration (1 × 10^8^ PFU/mL, multiplicity of infection [MOI] = 0.2) of the EATPs potently inhibited the infection of clinically isolated MDR P. aeruginosa (i.e., PA-154197, PA-139357) both *in vitro* and *in vivo*. Importantly, EATPs in combination with antibiotics displayed significantly improved performance against P. aeruginosa with antibiotic resistance that was caused by the abuse of antibiotics. Collectively, we clarified that EATPs suppress CRISPR-Cas system immunity to successfully replicate, dependent on Acr production. We also demonstrated the absolute inhibitory concentration of EATPs that was required to overcome CRISPR-Cas immunity and block bacterial growth. These results motivate the continued development of these promising, engineered bacteriophage therapeutics for the creation of next-generation antimicrobials.

## RESULTS

### Successful phage replication correlates with the competition between CRISPR and Acrs.

**FIG 1:**
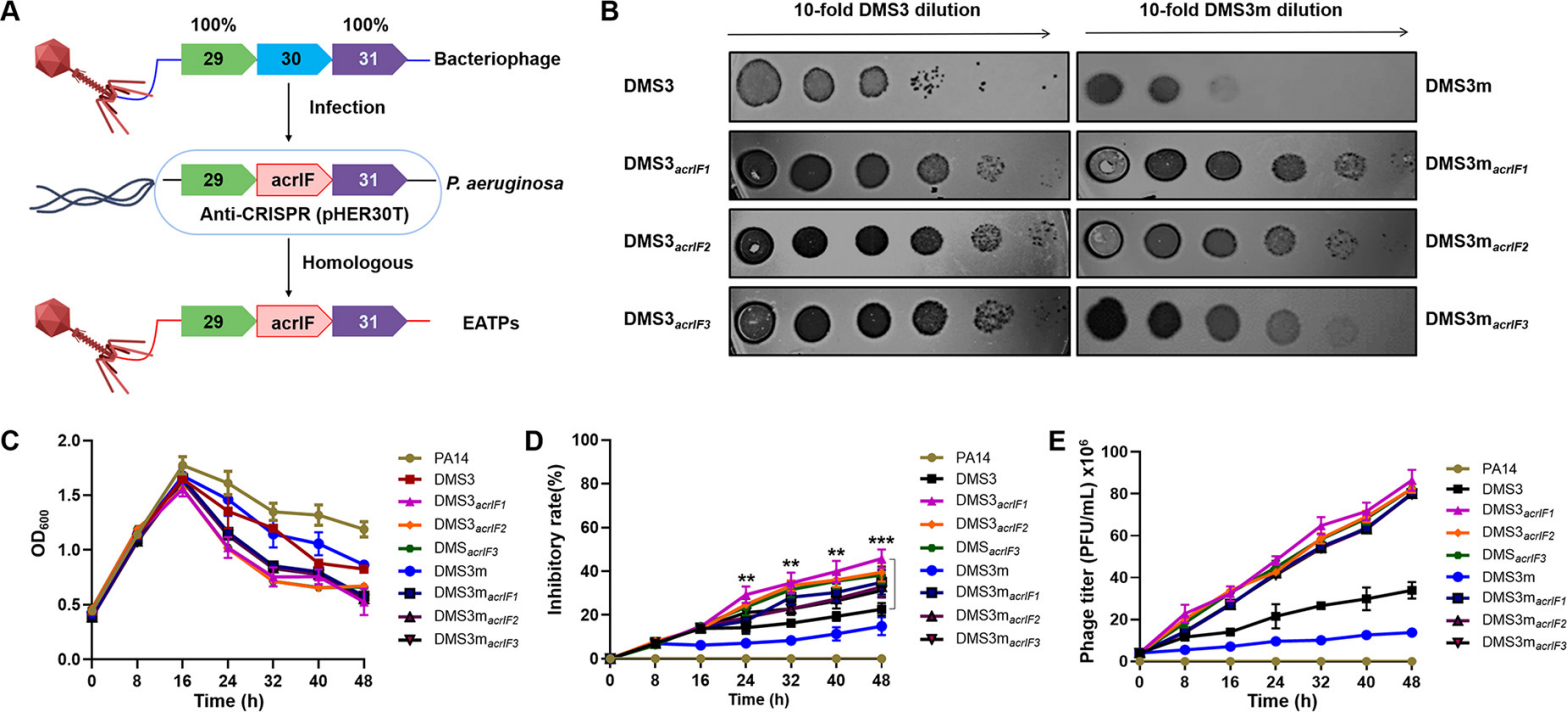
The evolutionary race between EATPs and CRISPR-Cas immunity. (A) Schematic illustration of the strategy to acquire EATPs. (B) Phage plaque assays were performed on PA14 (Phage: 1 × 10^7^ PFU/mL, PA14: 5 × 10^8^ CFU/mL, MOI = 0.02). The growth kinetics of PA14 (C) and the inhibitory rates (%) of EATPs (D) were evaluated within 48 h after treatment with 1 × 10^7^ DMS3, DMS3m phages, and EATPs (MOI = 0.02), respectively. (E) Phage titers were measured by PFU. Data were presented as mean ± standard error of the mean (SEM), determined from biological triplicates and analyzed via a one-way analysis of variance (ANOVA) compared against a control group (*, P < 0.05; **, P < 0.01; ***, P < 0.001; ****, P < 0.0001).

To determine whether Acrs contribute to the proliferation of phages by targeting CRISPR-Cas immunity, we constructed different types of engineered Acr-containing phages (EATPs) by inserting an Acr into DMS3 (CRISPR-insensitive phages) and DMS3m (CRISPR-sensitive phages) via homologous recombination (18) (Fig. 1A), amplified and characterized by PCR and agarose gel electrophoresis (Fig. S1). Next, an EATP plaque assay was performed on highly antibiotic-resistant PA14. Our data in Fig. 1B showed that the Acrs gene insertion into DMS3 enhanced the phage’s resistance to CRISPR-Cas immunity, counteracted the bacterial attack, and transformed the CRISPR-sensitive phages (DMS3m) into CRISPR-resistant phages. We further tested the PA14 growth kinetics (Fig. 1C) and the inhibitory rate (%) of the EATPs (Fig. 1D) from 0 h to 48 h after treatment with 1 × 10^7^ DMS3, DMS3m, or EATPs at an MOI of 0.02. The antibacterial effect of the EATPs was noticed after 16 h of phage treatment. These results suggested that the growth of PA14 can be inhibited only when the Acrs-phages achieve a certain concentration. That is, the production of Acrs needs to exceed the threshold of CRISPR immunity. Notably, the CRISPR-sensitive DMS3m took longer to get the same inhibitory effect as did the CRISPR-insensitive phage DMS3, suggesting that a certain quantity of Acrs is required to break potent CRISPR-Cas immunity. Likewise, we further detected the phage titers (Fig. 1E), and the results showed that the phage titers were negatively correlated with the growth of PA14 and positively correlated with the inhibitory rate. Collectively, these data suggest that successful phage replication may be dependent on the competition between CRISPRs and Acrs.

### EATPs inhibition of PA14 growth is dose-dependent.

**FIG 2:**
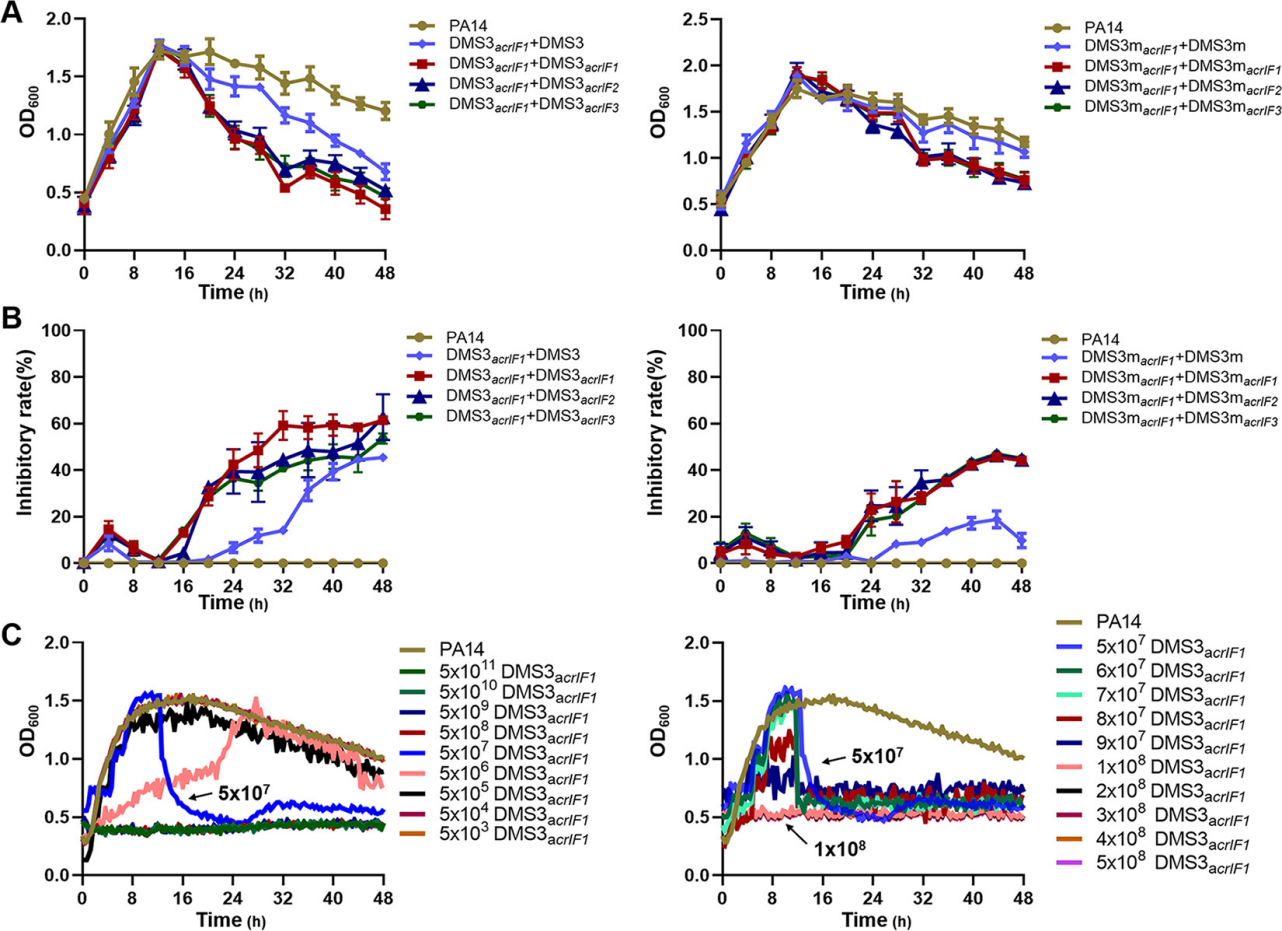
Acrs-mediated CRISPR-Cas inactivation requires a critical phage concentration. (A) PA14 was treated with DMS3*_acrIF1_*/DMS3m*_acrIF1_* for 12 h (1 × 10^7^ PFU, MOI = 0.02) and infected with DMS3*_acrIF1_/*DMS3 or DMS3m*_acrIF1_/*DMS3m for 36 h (1 × 10^7^ PFU, MOI = 0.02). Growth kinetics upon phage infection were continuously monitored from the initial DMS3*_acrIF1_* challenge to 48 h. (B) Inhibitory rates (%) of DMS3*_acrIF1_* on PA14 were evaluated within 48 h after treatment with DMS3*_acrIF1_* for 12 h and were infected with DMS3 or DMS3*_acrIF1_* for 36 h (1 × 10^7^ PFU, MOI = 0.02). (C) DMS3*_acrIF1_* of different titers was used to infect PA14 (5 × 10^8^ CFU/mL), and the growth kinetics upon phage infection were calculated by measuring the OD_600_ nm at 20 min intervals up to 48 h at 37°C with constant shaking. Data were presented as mean ± standard error of the mean (SEM), determined from biological triplicates and analyzed via a one-way ANOVA compared against a control group (*, P < 0.05; **, P < 0.01; ***, P < 0.001; ****, P < 0.0001).

Research has shown that Acr-containing phages suppress CRISPR immunity through a cooperation mechanism; initial infection by Acr phages triggers immunosuppression in the host, which helps to improve the efficiency of subsequent infections (28, 29). These results demonstrate that the initial titers of Acr-phages that are above a critical threshold value play a crucial role in inhibiting phage amplification to achieve a therapeutic effect. To confirm the specific critical threshold, we assessed the growth kinetics of PA14 (5 × 10^8^ CFU/mL) and the inhibitory rate (%) of phages to PA14 after treatment with DMS3*_acrIF1_* and infection with EATPs or DMS3 (no Acrs) for 36 h (1 × 10^7^ PFU, MOI = 0.02) (Fig. 2A and B). The results showed that the inhibitory rate of the EATPs was significantly increased by DMS3*_acrIF1/_*DMS3m*_acrIF1_* in combination with EATPs compared to controls (DMS3*_acrIF1_*/DMS3 or DMS3m*_acrIF1_*/DMS3m). These results confirmed that EATPs-mediated bacterial inhibition requires the production of Acrs to be above a threshold that prevents the CRISPR-Cas system immunity from breaking its barrier. Similarly, DMS3m needs to reach a higher threshold to inhibit the growth of PA14. DMS3*_acrIF1_* showed the strongest inhibitory effect on the growth of PA14; therefore, we evaluated the exact threshold of DMS3*_acrIF1_* by using different titers of DMS3_*acrIF1*_ infection with PA14 (Fig. 2C). We found that when the titers of the DMS3_*acrIF1*_ reached 5 × 10^7^ PFU/mL (MOI = 0.1), significant inhibition of PA14 occurred. Likewise, we further confirmed that the absolute inhibitory concentration is about 1 × 10^8^ PFU/mL (MOI = 0.2) by choosing a concentration gradient from 5 × 10^7^ PFU/mL (MOI = 0.1) to 5 × 10^8^ PFU/mL (MOI = 1). We found that the growth of PA14 was decisively suppressed above the absolute inhibitory concentration (1 × 10^8^ PFU/mL, MOI = 0.2). The above results indicated that 5 × 10^7^ PFU/mL of EATPs were required for phage replication in a CRISPR-harboring P. aeruginosa strain (PA14) and that 1 × 10^8^ PFU/mL of EATPs powerfully overcame CRISPR-Cas immunity and blocked bacterial growth, meaning that it can be used as the most effective dose for therapy P. aeruginosa infection.

### DMS3*_acrIF1_* completely inhibits the growth of clinically isolated CRISPR-harboring P. aeruginosa.

**FIG 3:**
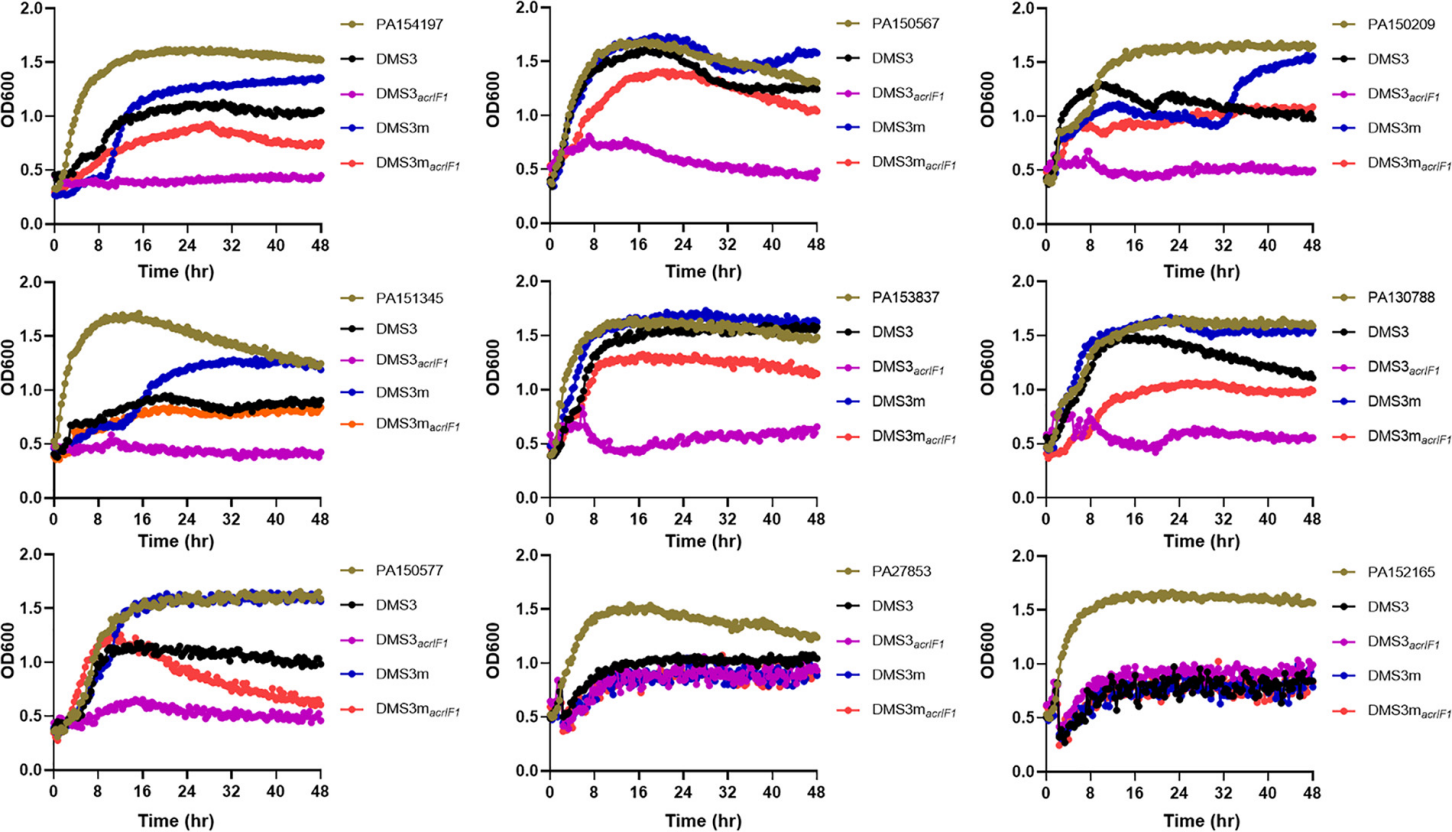
DMS3*_acrIF1_* mitigates clinically isolated P. aeruginosa infection. The clinically isolated P. aeruginosa (5 × 10^8^ CFU/mL) were treated with DMS3*_acrIF1_* or control phages for 48 h (1 × 10^8^ PFU/mL, MOI = 0.2). The growth kinetics of P. aeruginosa upon phage infection were obtained by measuring the OD_600_ nm at 20 min intervals up to 48 h at 37°C with constant shaking. Data were presented as mean ± standard error of the mean (SEM), determined from biological triplicates and analyzed via a one-way ANOVA compared against a control group (*, P < 0.05; **, P < 0.01; ***, P < 0.001; ****, P < 0.0001).

To further characterize whether DMS3*_acrIF1_* has a broad-spectrum role in inhibiting P. aeruginosa infection, we analyzed the growth kinetics (DMS3*_acrIF1_* and DMS3m*_acrIF1_*; 1 × 10^8^ PFU/mL, MOI = 0.2) of multiple clinical isolates of P. aeruginosa (CRISPR-Cas system has been identified; Table S1) and showed that DMS3*_acrIF1_* had an overwhelming dynamic repression of bacterial growth (Fig. 3). CRISPR-harboring P. aeruginosa was completely incapable of breaking the anti-CRISPR defenses when DMS3*_acrIF1_* reached the absolute inhibitory concentration (1 × 10^8^ PFU/mL, MOI = 0.2). From a mechanistic view, Acrs confer upon DMS3m*_acrIF1_* a partially defensive ability against CRISPR-Cas immunity, illustrating that sufficient Acr production is necessary for successful phage therapy. Overall, these results indicated that DMS3m*_acrIF1_* had a broad-spectrum inhibitory effect on P. aeruginosa, offering promise for the treatment of clinically isolated P. aeruginosa infections.

### DMS3*_acrIF1_* therapeutically controls clinical MDR P. aeruginosa infection.

**FIG 4:**
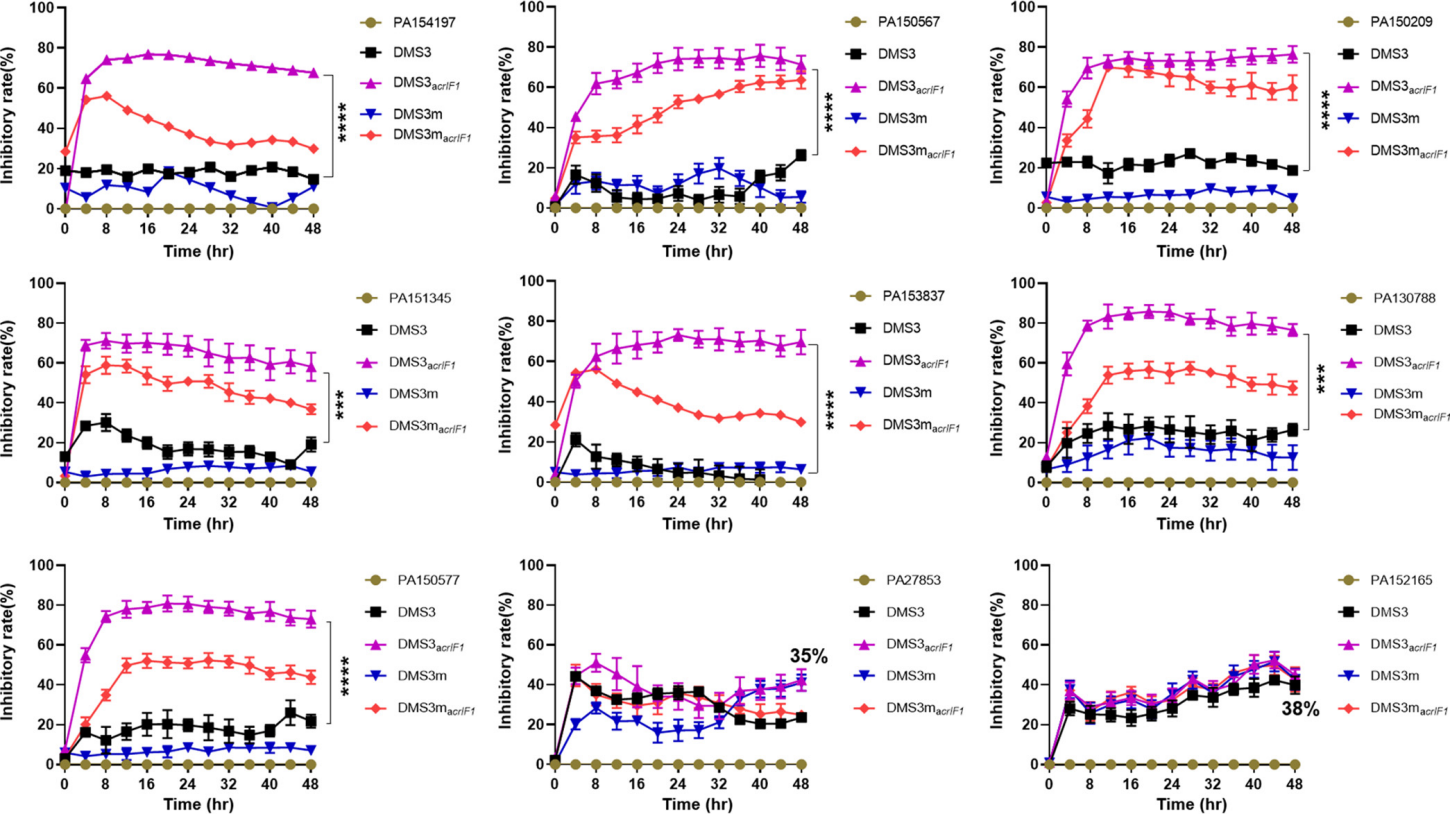
DMS3*_acrIF1_* potently inhibits clinically isolated P. aeruginosa infection. The clinically isolated P. aeruginosa strains were treated with DMS3*_acrIF1_* or control phages for 48 h (1 × 10^8^ PFU/mL, MOI = 0.2). The inhibitory rate (%) of DMS3*_acrIF1_* on P. aeruginosa was evaluated upon phage infection by measuring CFU at 4 h intervals up to 48 h at 37°C with constant shaking. Data were presented as mean ± standard error of the mean (SEM), determined from biological triplicates and analyzed via a one-way ANOVA compared against a control group (*, P < 0.05; **, P < 0.01; ***, P < 0.001; ****, P < 0.0001).

To further characterize whether DMS3*_acrIF1_* can be used to treat clinical P. aeruginosa infection, we analyzed the inhibitory rate (%) of DMS3*_acrIF1_* (DMS3*_acrIF1_* and DMS3m*_acrIF1_*; 1 × 10^8^ PFU/mL, MOI = 0.2) to clinical isolates of P. aeruginosa and showed that the inhibitory rate of DMS3*_acrIF1_* is maintained at 60 to 80% (Fig. 4). Additionally, although neither the DMS3*_acrIF1_* nor the DMS3 phages completely inhibited the growth of CRISPR-harboring P. aeruginosa (PA27853 and PA152165), they still exhibited an inhibitory effect of approximately 35%. The inhibitory rate (%) of DMS3_*acrIF1*_ on CRISPR-harboring P. aeruginosa was 40% higher than that of DMS3 without Acrs, suggesting that the given Acrs provide an inhibition rate of approximately 30% by modulating the CRISPR response. Similarly, Acrs significantly increased the approximately 40% inhibitory rate of DMS3m*_acrIF1_* compared to DMS3m, indicating that DMS3*_acrIF1_* could be used as an effective strategy for controlling clinically MDR P. aeruginosa infection. Collectively, these results indicated that engineered phages containing anti-CRISPR can act as an alternative or a supplementary treatment for P. aeruginosa infection.

### DMS3*_acrIF1_* controls respiratory infections caused by MDR P. aeruginosa infection.

**FIG 5:**
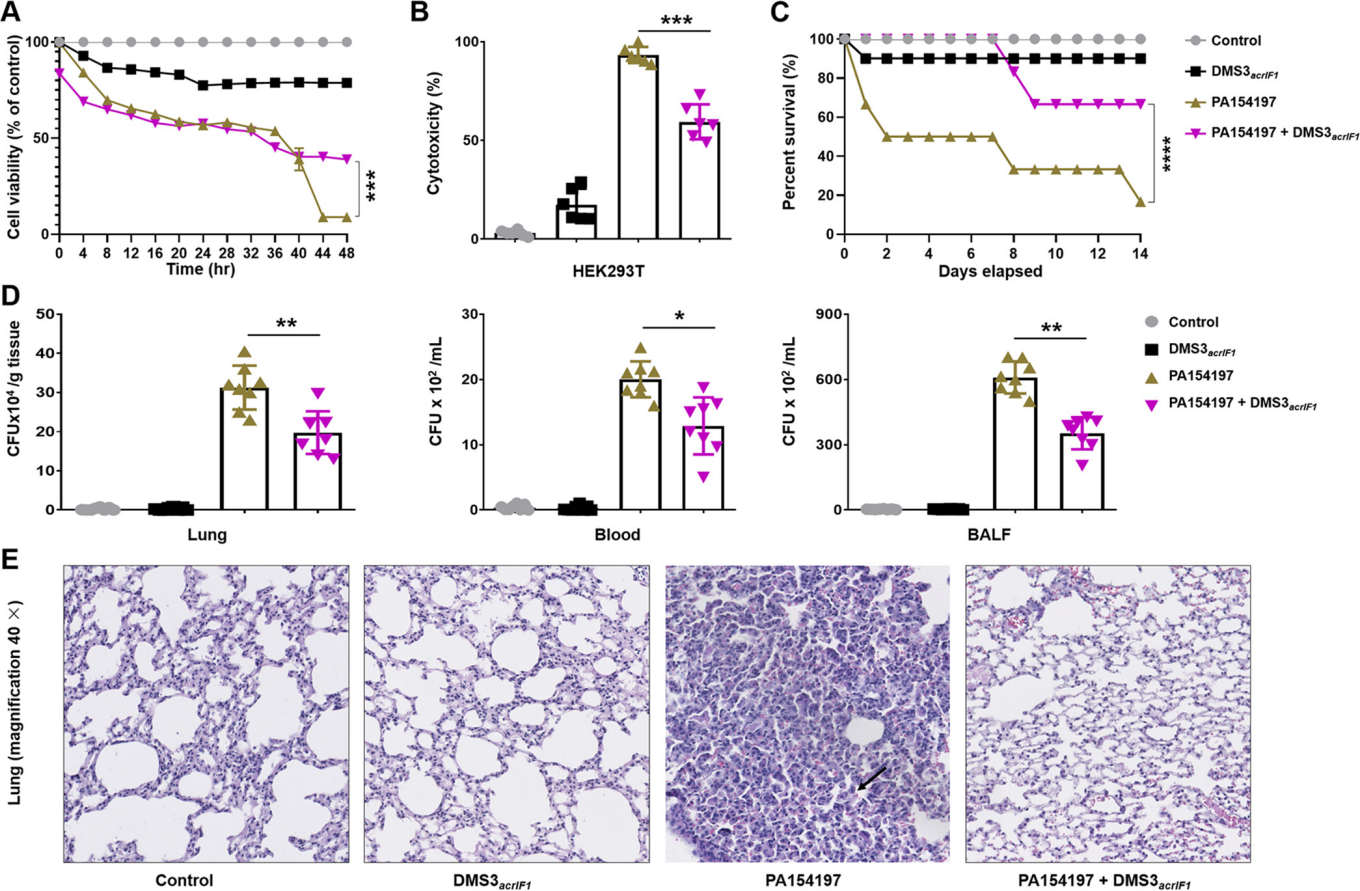
DMS3*_acrIF1_* mitigates tissue injury and improve survival. HEK293T cells grown to subconfluence (1 × 10^5^ cells in a 96-well plate) were infected with PA154197 for 1 h (1 × 10^6^ CFU/mL, MOI = 10) and treated with 2 × 10^5^ PFU/mL DMS3*_acrIF1_* (MOI = 0.2). Cell viability (A) and toxicity (B) were measured by MTT and LDH assays in HEK293T. C57BL/6N mice (*n* = 10, both sexes) were anesthetized with ketamine (45 mg/kg), intranasally infected with 6 × 10^6^ CFU/25g clinically isolated MDR P. aeruginosa PA154197 for 2 h, and intraperitoneally injected with 1.2 × 10^6^ PFU/mL DMS3*_acrIF1_* (MOI = 0.2). Survival was monitored within 14 days postinfection and is represented by Kaplan-Meier survival curves (C). Bacterial burden (D) and histological analysis (E) were also measured. Data were presented as mean ± standard error of the mean (SEM), determined from biological triplicates and analyzed via a one-way ANOVA compared against a control group (*, P < 0.05; **, P < 0.01; ***, P < 0.001; ****, P < 0.0001).

Phages are natural symbionts of humans and do not adversely affect eukaryotic cells, even at high titers (30). To assess the safety of DMS3*_acrIF1_*, HEK293T cells (approximately 1 × 10^5^ cells) were infected with clinically isolated MDR P. aeruginosa PA154197 in a 24-well plate for 1 h (1 × 10^6^ CFU P. aeruginosa, MOI = 10), and then treated with 2 × 10^5^ PFU/mL DMS3*_acrIF1_* (MOI = 0.2). We examined cell viability (Fig. 5A) and toxicity (Fig. 5B) by MTT and LDH cytotoxicity assay in HEK293T. The results showed that DMS3*_acrIF1_* significantly increased the survival of HEK293T while exhibiting low toxicity, suggesting safe and efficient features. The biggest challenges of phage therapy is probably the overcoming of pharmacological barriers, including biofilm matrix-mediated penetration barriers, immune system-mediated trapping (by neutrophils), mononuclear phagocyte system (MPS)-mediated clearance, and others (31–33). A variety of injection routes have been explored to disarm the body’s innate defense barriers (33, 34). Intraperitoneal injection, allowing for the rapid delivery of high titers of phage into the blood circulation, shows potent therapeutic effects on penetrating the pharmacological barriers for treating respiratory infections or septicemia (35–38). A single intraperitoneal injection of phages provides 100% protection from K. pneumoniae-mediated respiratory infections to animals (38). To test whether DMS3*_acrIF1_* specifically inhibits bacterial invasion *in vivo*, we employed a mouse respiratory infection model to explore the therapeutic effects of DMS3*_acrIF1_* phages. C57BL/6N mice were intranasally infected with 6 × 10^6^ CFU/25 g of clinically isolated MDR P. aeruginosa PA154197 for 2 h. Then, the mice were intraperitoneally injected with 1.2 × 10^6^ PFU/mL DMS3*_acrIF1_* (MOI = 0.2). We analyzed the survival curve (Fig. 5C) and bacterial burdens (Fig. 5D), which showed that DMS3*_acrIF1_* significantly increased the survival rates and decreased the bacterial burdens of PA154197 in the mouse lung homogenate, blood, and bronchoalveolar lavage fluid (BALF) versus controls without the DMS3*_acrIF1_*. Furthermore, DMS3*_acrIF1_* introduction significantly attenuated tissue damage and inflammatory responses in mice by H&E staining (Fig. 5E; Fig. S2). Collectively, these results demonstrated that the DMS3*_acrIF1_* displayed high safety and strong efficacy for inhibiting the infection caused by MDR P. aeruginosa *in vitro* and *in vivo*. Altogether, our analysis supports that EATPs can suppress CRISPR-Cas immunity and soothe the disease progression of a P. aeruginosa infection.

### Synergistic phage-antibiotic combinations attenuates antibiotic resistance.

**FIG 6:**
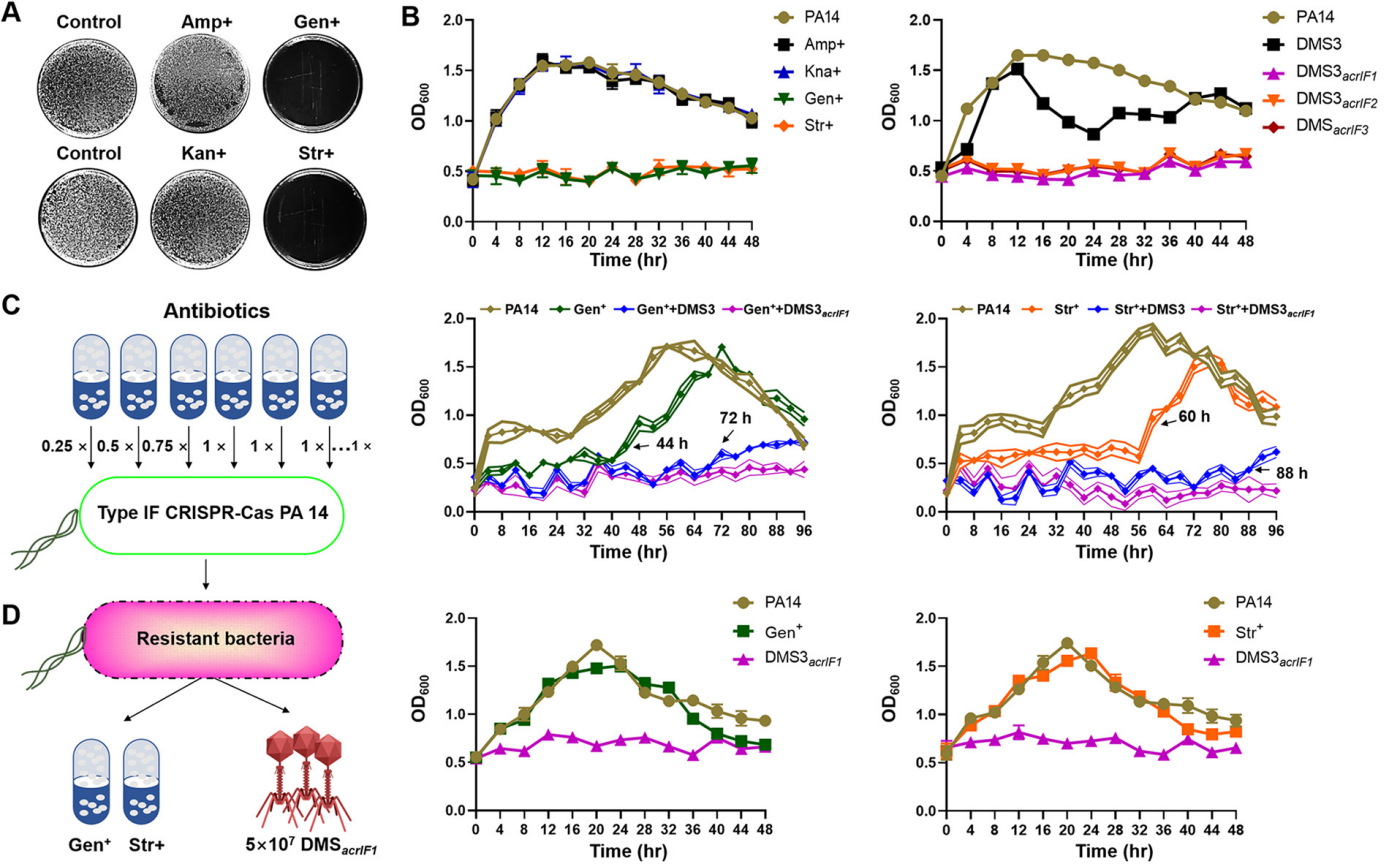
DMS3*_acrIF1_* ameliorates P. aeruginosa antibiotic resistance. (A) PA14 (OD_600_ = 0.5) was evenly spread on a plate containing different antibiotics (Amp^+^: 100 μg/mL, Kan^+^: 100 μg/mL, Gen^+^: 100 μg/mL, Str^+^: 100 μg/mL), and antibiotic resistance was tested by observing the growth of bacteria on the plate. (B) PA14 (5 × 10^8^ CFU/mL) was cultured in lysogeny broth (LB) containing different antibiotics (Amp^+^: 100 μg/mL, Kan^+^: 100 μg/mL, Gen^+^: 100 μg/mL, Str^+^: 100 μg/mL) or treated with 1 × 10^8^ PFU/mL EATPs/DMS3 (MOI = 0.2). The growth kinetics of PA14 were continuously monitored at 4 h intervals up to 48 h at 37°C with constant shaking. (C) The left panel is a schematic diagram of the antibiotic resistance experiment. PA14 (PA14: 5 × 10^8^ CFU/mL) was treated with gradient concentrations of antibiotic (Gen^+^ or Str^+^), gradient concentrations of antibiotic and DMS3 (MOI = 0.02) (Gen^+^/Str^+^ + DMS3) or Gen^+^/Str^+^ + DMS3*_acrIF1_*. The growth kinetics of PA14 were measured. (D) PA14 that acquired antibiotic resistance were picked out and grown to the mid-logarithmic phase (OD_600_ = 0.4 to 0.6) in LB at 37°C with 220 rpm shaking. The growth kinetics of the acquired antibiotic resistance of PA14 were measured after treatment with DMS3*_acrIF1_* (MOI = 0.2) or with 100 μg/mL Gen^+/^Str^+^ antibiotics. Data were presented as mean ± standard error of the mean (SEM), determined from biological triplicates and analyzed via a one-way ANOVA compared against a control group (*, P < 0.05; **, P < 0.01; ***, P < 0.001; ****, P < 0.0001).

**FIG 7:**
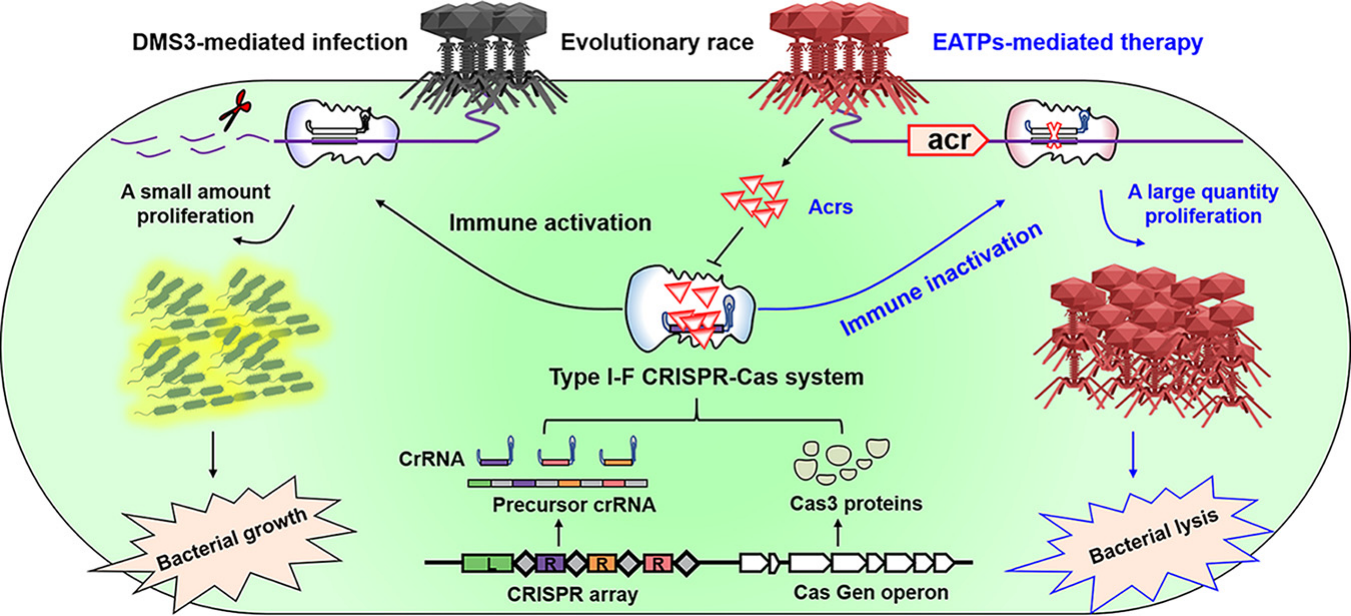
Schematic illustration of EATPs in suppressing P. aeruginosa infection. Normally, phage infection activates CRISPR-Cas adaptive immunity, which blocks phage replication through the cleavage of phage genomes, resulting in only a small amount of proliferation of phages, eventually preserving the bacterial homeostasis. EATPs suppress the activity of CRISPR-Cas systems to protect their associated phage genomes by producing Acrs, resulting in a large quantity proliferation of phages, eventually, the bacteria are lysed. EATPs have great potential for treating antibiotic-resistant P. aeruginosa infection by lysing bacteria.

Drug resistance is recognized as a major public health threat and severely hinders the effects of antibiotics (39). It has been well-documented that combining phages and antibiotics may help to counteract antibiotic resistance and thus serve as an efficient therapy of bacterial infection (40, 41). Phage-antibiotic synergy (PAS) has been used to treat a variety of bacterial infections, including Escherichia coli (ciprofloxacin and phage ECA2) (42, 43), P. aeruginosa (phage LUZ7 and streptomycin) (44) and Staphylococcus aureus (phage SAP-26 and rifampicin) (45, 46). However, whether DMS3*_acrIF1_* can alleviate antibiotic resistance is unclear. Thus, we analyzed high antibiotic resistance PA14 with conventional antibiotics, including Ampicillin (Amp^+^), Kanamycin (Kan^+^), Gentamicin (Gen^+^), and Streptomycin (Str^+^) (Fig. 6A), and found that PA14 intrinsically carried antibiotic resistance to Amp^+^ and Kan^+^ but not to Gen^+^ and Str^+^. Therefore, either Gen^+^/Str^+^ or 1 × 10^8^ PFU/mL DMS3*_acrIF1_* showed a strong inhibitory effect on PA14 (Fig. 6B). To more clearly understand the DMS3*_acrIF1_*-antibiotic synergy, we evaluated the effects of a lower concentration of a DMS3/DMS3*_acrIF1_* combination with Gen^+^/Str^+^ on PA14 infection (PA14: 2 × 10^8^ CFU/mL; DMS3*_acrIF1_*: 4 × 10^6^ PFU/mL, MOI = 0.02; the low concentration of DMS3/DMS3*_acrIF1_* has been shown to not completely inhibit the growth of PA14 in Fig. 1). The results showed that antibiotic resistance can be induced by adding gradient concentrations of antibiotics with increasing incubation times (Gen^+^: 44 h, Str^+^: 60 h). Importantly, the low concentration of DMS3 delayed the emergence of PA14 resistance (Gen^+^:72 h, Str^+^: 88 h), but the low concentration of DMS3*_acrIF1_* rescued the Gen^+^ and Str^+^ antibiotic resistance caused by persistent antibiotic induction within 96 h, which may be related to the stronger antibacterial ability of DMS3*_acrIF1_* than DMS3 (Fig. 6C; Fig. S3A). These results indicated that DMS3*_acrIF1_* enable the long-term suppression of bacterial growth *in vitro* and prevent the appearance of antibiotic resistance. Next, we picked out PA14 that acquired resistance to Gen^+^ and Str^+^ and tested the growth kinetics of PA14 and the inhibitory rate (%) of either DMS3*_acrIF1_* (MOI = 0.2) or an antibiotic (100 μg/mL) to tolerated PA14 (Fig. 6D; Fig. S3B). We found that DMS3*_acrIF1_* suppressed the growth of MDR PA14 with a potent inhibition rate (approximately 40%), whereas the antibiotic alone did not work. Together, these findings indicate that DMS3*_acrIF1_* in combination with antibiotics have a strong effect on inhibiting antibiotic resistance and may be considered an alternative therapy by which to treat infections of MDR bacteria (Fig. 7).

## DISCUSSION

In this paper, we present a novel approach to suppressing P. aeruginosa antibiotic resistance and halting infection with newly engineered AcrIF gene-containing phages, EATPs. Our data suggested that these EATPs may be used to prevent or attenuate bacterial infection caused by either high antibiotic resistance PA14 strains or clinically isolated MDR P. aeruginosa, representing a potential alternative, anti-infective strategy by which to control P. aeruginosa infection. Importantly, our findings demonstrated that EATPs combined with antibiotics may serve as a novel treatment strategy by which to curb antibiotic resistance. These findings addressed the concern about the treatment of antibiotic-resistant bacterial infection. Our study provides experimental evidence that CRISPR-Cas inhibitors act as potential anti-infective means to halt intractable bacterial infection.

Phages, as the natural predators of bacteria, kill bacteria by causing lysis effects (47). There are several advantages of phage therapy, including replication at the infection site and the fact that it is typically harmless not only to the host organism but also to other beneficial bacteria, such as the gut flora, which reduces the chances of opportunistic infections (48). Phage therapeutics have a highly specific and targeted killing mechanism that only attacks the target bacterial strains, allowing them to spread in nearby areas and cause bacterial lysis by modulating the number of phages to the inundation threshold (the concentration of free phage required for reducing bacterial loads) (31). We observed that the DMS3 natural phages do not inhibit the growth of PA14 at a MOI of 0.02 after 16 h of infection (Fig. 1), indicating that higher phage concentrations are necessary to reach the inundation threshold or to break bacterial defenses, increasing therapeutic risk. Natural evolution has shown that phages can halt bacterial infections, but they lack control and are slow to progress (49). Phage inhibition of PA14 proliferation manifested 24 h after infection, when the bacteria entered the death phase (Fig. 2). We clarified that phages cannot substantially control PA14 infection if it occurs in an *in vivo* environment that is more prone to bacterial growth.

Bacteria and phages have been in an evolutionary arms race (30, 50). Bacteria have evolved a range of defensive weapons to prevent phage infection, and, among those, the CRISPR-Cas systems represent a paradigm shift of bacterial immunity in that they provide a clinical difficulty in the control of infection, even though they have improved gene-editing technology for humans (51, 52). As a result, phage therapy strategies often require a large number of phages that may be composed of multiple unrelated phages, which may trigger the immune system to overreact or cause an immune imbalance (16, 53). CRISPR-Cas systems provide adaptive immunity against an invasion of phages by cutting phage nucleic acids and/or dampening their invasion and pathogenic effects (54). The crRNA-guided cascade complex and the recruitment of Cas3 are the keys to initiate Type I CRISPR-Cas immunity (55, 56). Currently, Type I-F, Type I-E, and Type I-C have been widely found in P. aeruginosa. Correspondingly, Type I-F anti-CRISPR genes (AcrF1 to AcrF7, AcrF1 to AcrF12), Type I-E anti-CRISPR genes (AcrE1 to AcrE7, AcrE9), and Type I-C anti-CRISPR genes (AcrIC1, AcrIC3 to IC8) have also been identified (16, 27, 55, 57–59). Mechanically, Acrs inhibit CRISPR-Cas immunity mainly by directly inhibiting DNA binding, including blocking the hybridization of target DNA with crRNA guides (AcrF1, AcrIF8, AcrIF9, and AcrIF14) and blocking PAM binding sites on double-stranded DNA (AcrIF2, AcrIF6, AcrIF7, AcrIF10, and AcrIF11). AcrIE1 and AcrIF3 prevent Type I CRISPR-Cas immunity by directly binding to Cas3 (57). Apparently, Acrs is one of the important strategies that phages employ to evade bacterial arsenals (Fig. 3 and 4). We confirmed that the engineered anti-CRISPR (AcrIF1, AcrIF2, and AcrIF3) containing phages significantly enhanced the antibacterial activity of DMS3 and DMS3m, even below the threshold.

Traditionally, phage therapy is a naturally occurring process, is uncontrollable, and relies chiefly on the characteristics of the phage itself (11, 60, 61). In contrast, harnessing novel technology advances beyond natural evolution and makes it possible to engineer specific phages to achieve long-term and broad-spectrum suppression of bacterial immune defenses and drug resistance (62). Acr-bearing phages block the host CRISPR-Cas immune systems with a first phage to allow a second Acr-phage to successfully replicate (28). The production of Acrs by a phage genome is a determinant of successful phage infection (29). These results demonstrated that phage cooperation suppresses bacterial CRISPR-Cas immunity and mediated bacterial lysis, illustrating the potential of engineered anti-CRISPR-Cas phages in overcoming MDR bacterial infection. However, it remains untested whether these engineered Acr gene-containing phages (EATPs) can be used to counteract bacterial infections, particularly those caused by MDR strains, which are increasingly common in clinics. We observed that EATPs strongly overcame CRISPR-Cas immunity and blocked bacterial growth when the initial concentration of phages surpassed the critical threshold and achieved the absolute inhibitory concentration (Fig. 2). These data demonstrated that the successful replication of phages for the inhibition of bacterial growth is Acr-dependent and that this is achievable by the synergy of phage flora. Additionally, our analyses revealed for the first time that the EATPs contribute to the suppression of the growth of clinical isolates of MDR P. aeruginosa *in vitro* and *in vivo* (Fig. 3 and 5). We confirmed that the tipping points of phage concentration are necessary for regulating the density of the bacterial population.

P. aeruginosa acquires drug resistance in its lifetime through various mechanisms, including intrinsic and adaptive drug resistance, resulting in stellar resistance to multiple antibiotics, including aminoglycosides, quinolones, polymyxins, and β-lactams (63). There is an urgent need to develop alternative therapeutic approaches to treat P. aeruginosa infections, especially after all treatment options are exhausted (64). Phages are selective for particular bacterial strains, as opposed to conventional chemical antibiotics, most of which exhibit broad-spectrum activity, which contributes to antibiotic resistance development (65, 66). Antibiotics interfere with bacterial metabolism in favor of successful phage infection (47, 67). Phage-antibiotic combination treatments can be effective against antibiotic resistance by retaining their antibacterial pharmacodynamic activity, including bacterial biofilms and persister cells (67). We confirmed that the EATPs in combination with antibiotics effectively counter antibiotic resistance, suggesting their potential to serve as an alternative therapy by which to treat infections of antibiotic-resistant bacteria (Fig. 6).

In summary, we have pioneered a proof of concept of engineered Acrs-containing phages, and we have unveiled their safety and efficacy in inhibiting P. aeruginosa infection, which may represent an innovative alternative therapeutic strategy for patients, especially in severe infections by multidrug resistant strains. Having confirmed their antibacterial activities in cells, we demonstrated that EATPs alleviate bacterial burdens, antibiotic resistance, and histological damage, leading to longer survival of infected mice. Further, no major organ toxicities were observed in the phage treatment group, suggesting that this therapeutic alternative may be safe in clinics. Mechanistically, our studies also indicate that a sufficient initial concentration of phages may trigger the production of Acrs to counter the function of CRISPR/Cas immune systems, which is necessary for successful EATPs therapeutic effects. Our work provides a framework in which to continue the development of anti-CRISPR phages to treat intractable Pseudomonas disease and other bacterial infections (Fig. 7).

## MATERIALS AND METHODS

### Ethics statement.

This study was carried out in accordance with the recommendations in the Guide for the Care and Use of Laboratory Animals of the National Institutes of Health. The protocols were approved by the Institutional Animal Care and Use Committee at the University of North Dakota. All of the animal experimental procedures were performed under anesthesia that was induced and maintained with ketamine hydrochloride and xylazine, and all efforts were made to minimize animal suffering.

### Animals.

C57BL/6N mice (6 to 8 weeks) were purchased from Harlan Laboratory. Mice were maintained in the animal facility at the University of North Dakota for 2 weeks before the experiment. Both males and females were used with random grouping. All animal studies were approved by the University of North Dakota Institutional Animal Care and Use Committee and performed following the animal care and institutional guidelines.

### Cell lines.

HEK293T were obtained from American Type Culture Collection (ATCC, Manassas, VA) and cultured following the manufacturer’s instructions (68).

### Bacterium.

The wild-type P. aeruginosa strain PA14 (PA14) was a gift from Steve Lory (Department of Molecular Genetics, Harvard Medical School). Clinically isolated pathogenic P. aeruginosa strains were obtained from Aixin Yan (School of Biological Sciences, the University of Hong Kong) (Table S1) (69). Bacteria were grown to the mid-logarithmic phase (OD_600_ = 0.4 to 0.6) in lysogeny broth (LB) at 37°C with 220 rpm shaking. The number of bacteria was determined by measuring the OD_600_ nm value (1 OD = 1 × 10^9^ cells/mL).

### Phages.

Wild-type P. aeruginosa phage DMS3 and its mutant DMS3m (DMS3_100%_), Pseudomonas phage JBD30 (*AcrIF1*), Pseudomonas phage D3112 (*AcrIF2*), and Pseudomonas phage JBD88a (*AcrIF3*) were obtained from George A. O'Toole (Dartmouth College) (70). Grown P. aeruginosa were lysed by phages at 37°C with 220 rpm shaking for 48 h, and the phage supernatant was collected by centrifugation for 15 min at 12,000 r/min. The number of phages was quantified by the Plaque-Forming Unit (PFU) (71). Briefly, PFU was carried out on plates containing LB with 1.5% agar and another layer (0.3%) containing a bacteria and phage mixture on the top. 300 μL of P. aeruginosa (OD_600_ = 0.4) were mixed with 100 μL of phages (Serially diluted 10^−3^ to 10^−7^). Then, 0.3% molten soft LB agar was added to the mixture and was poured on top of the hard agar layer. Plates were incubated overnight at 37°C, and the number of phages was determined using the expression: plaque-forming unit/mL × dilution factor.

### Engineered Acr gene-containing phages (EATPs).

DMS3 is a CRISPR-insensitive phage that contains the Type I-E anti-CRISPR gene (*gene 30*, encoding AcrIE3). The most common intact CRISPR-Cas system found in P. aeruginosa is the Type I-F CRISPR-system, which provides the main immune defenses for clinically isolated P. aeruginosa (25, 26, 72, 73). In contrast, studies have shown that AcrIE3, as a weak inhibitor, does not target the Type I-F CRISPR-system (28). Therefore, we attempted to tackle clinically isolated MDR P. aeruginosa by replacing the gene 30 (AcrIE3) of DMS3/DMS3m with potent Type I-F anti-CRISPR genes *AcrIF1* (NCBI Reference Sequence: YP_007392342.1), *AcrIF2* (NCBI Reference Sequence: NP_938237.1), and *AcrIF3* (NCBI Reference Sequence: YP_007392440.1) (18). DMS3m contains five point mutations in gene 42, creating a 100% match to the Type I CRISPR-system crRNA, which is easily targeted by Type I-F CRISPR-systems; thus, DMS3m is a CRISPR-sensitive phage (18). DMS3m was used as a control to explore the interaction of the Type I-F CRISPR-system and the Type I-F Acrs. Various phages bearing the Acr genes were generated by homologous recombination (18). Briefly, the pHERD30T (p30T, Escherichia-Pseudomonas shuttle vector pHERD30T, NCBI accession: EU603326, generous gift from Hongwei D. Yu) (74) was linearized by HindIII (2900) and NcoI (2967), and it was purified and extracted with a GeneJET Gel Extraction Kit (K0691: Thermo Fisher). The homology arms from the DMS3 (*gene 30*) genome upstream and downstream (*gene 29*: 578 bp and *gene 31*: 525 bp, NotI and *Pae*I restriction sites were included in the downstream *gene 31* forward primer) (Table S2), were amplified (Fig. S1A) and assembled into linearized pHERD30T using Gibson assembly (A13288, Life Technologies Corporation, Gaithersburg, MD). Recombined *gene* p30T-29/30 (gentamicin resistance) plasmid was picked through LB plates containing gentamicin, confirmed by agarose gel electrophoresis (Fig. S1B), and extracted with a GeneJET Plasmid Miniprep Kit (K0503, ThermoFisher Scientific). Different types of *Acr* genes (*AcrIF1*, *AcrIF2*, *AcrIF3*) that contained NotI and *Pae*I restriction sites at both ends of the gene were amplified from a Pseudomonas phage by PCR, digested by NotI and *Pae*I, and ligated with p30T*-gene 29*-*gene 31* by T4 DNA Ligase (M0202, NEB). The final ligation mixture was transfected into competent cell E. coli DH5-α (C2987I, NEB). Recombined p30T*-gene 29*-Acr-*gene 31* plasmid was picked, confirmed by agarose gel electrophoresis, and extracted with a GeneJET Plasmid Miniprep Kit (K0503, ThermoFisher Scientific). Next, the purified and quantitated plasmids bearing Acr genes were transfected into PA14 as described previously (75). PA14 containing p30T*-gene 29*-Acr-*gene 31* was infected with DMS3 and DMS3m to allow the phages to acquire Acr by homologous recombination (Fig. S1C). Phages that successfully acquired Acr lysis PA14 would form plaques. These plaques contained several types of EATPs (DMS3*_acrIF1_*, DMS3*_acrIF2_*, DMS3*_acrIF3_*; DMS3m*_acrIF1_*, DMS3m*_acrIF2_*, DMS3m*_acrIF3_*), were selected and purified three times by replating on wild-type PA14, and confirmed by PCR (18) (Fig. S1D).

### Plaque assays.

Plaque assay was carried out on plates containing a first layer of 1.5% agar. 300 μL of P. aeruginosa were mixed with 0.3% molten soft LB agar that was poured on top of the hard agar layer. Next, 3 μL of serially diluted phages were spotted on the plates and were then incubated overnight at 37°C (76). Plaques were formed by phage replication and by lysis of the bacterial host. The images were captured on a ChemiDoc Imaging System (BIO-RAD).

### Analysis of P. aeruginosa growth kinetics and inhibitory rate (%).

Phages of different titers were mixed with P. aeruginosa in a 96-well plate and incubated at 37°C with constant shaking for 48 h or 96 h. The growth kinetic curves of P. aeruginosa were obtained by taking OD_600_ nm measurements using a BioTek Synergy H1 microplate reader at 20 min, 4 h, or 8 h intervals. The inhibitory rate (%) of phages on P. aeruginosa replication was assessed by measuring CFU. Briefly, phages of different titers were mixed with P. aeruginosa in 1.5 mL Eppendorf tubes. The precipitate was collected by centrifugation for 10 min at 4,000 r/min (bacteria in pellet, phage in supernatant). Bacteria were serially diluted in multiples of 10. 100 μL of bacterial solution were plated evenly on plates containing LB with 1.5% agar. Colony forming units (CFUs) were subsequently counted for each plate. The inhibitory rate (%) of P. aeruginosa was quantified using the expression: ([Control group counted CFU – experimental group counted CFU]/control group counted CFU) × 100%.

### Construction of cells and animal infection models.

HEK293T cells grew to subconfluence (1 × 10^5^ cells in a 96-well plate) and were infected with clinically isolated MDR P. aeruginosa PA154197 at multiplicity of infection of 10:1 for 1 h (1 × 10^6^ CFU P. aeruginosa in a 96-well plate). Then, the cells were treated with 2 × 10^5^ PFU/mL DMS3*_acrIF1_* (MOI = 0.2). C57BL/6N mice were anesthetized with ketamine (45 mg/kg), intranasally infected with 6 × 10^6^ CFU/25 g of clinically isolated MDR P. aeruginosa PA154197 for 2 h, and subjected to an intraperitoneal injection of 1.2 × 10^6^ PFU/mL DMS3*_acrIF1_* (MOI = 0.2). Survival was monitored within 14 days, postinfection. The bacterial burden was monitored, and a histological analysis was carried out after completing the survival curve test.

### 2,5-diphenyl-2H-tetrazolium bromide (MTT) assay.

Cells were stained with trypan blue, and the number of viable cells was quantified through a cell-counting plate (adjusted to 1 × 10^5^ cells/well). P. aeruginosa (1 × 10^6^ CFU/mL) was added to the cells in a 96-well plate, incubated at 37°C/5% CO_2_ for the listed time, and then treated with 2 × 10^5^ PFU/mL DMS3*_acrIF1_* (MOI = 0.2). 20 μL of MTT solution (M6494; Thermo Fisher Scientific) (5 mg/mL, 0.5% MTT) were added to each well and were allowed to culture for 4 h. Stop solution was added to dissolve the formazan product. The cell viability was quantified by an absorbance measurement at 560 nm using a spectrometric plate reader (77).

### Cell cytotoxicity assay.

Cell cytotoxicity was evaluated using an LDH-Cytotoxicity Assay Kit (ThermoFisher Scientific) according to the manufacturer’s instructions. Briefly, HEK293T cells (1 × 10^5^ cells/well) were infected with P. aeruginosa (1 × 10^6^ CFU/mL) for 1 h, subsequently added to 2 × 10^5^ PFU/mL DMS3*_acrIF1_* (MOI = 0.2), and incubated at 37°C/5% CO_2_ for 12 h. The absorbance of all samples was obtained at 495 nm using a spectrometric plate reader, and the cytotoxicity percentage of the HEK293T cells was calculated (78).

### Bacterial burden assay.

Mouse lung tissue was homogenized with PBS, blood was obtained by cardiac blood sampling on a sacrificed mouse, and bronchoalveolar lavage fluid (BALF) was obtained through alveolar lavage as previously described at 14 days after bacterial or PBS infection (79). All samples were then diluted to different concentrations in PBS before being evenly distributed on LB dishes. The dishes were cultured in a 37°C incubator overnight, and the number of bacteria was counted. The experiment was performed in triplicate.

### Histological analysis.

Mouse heart, liver, spleen, kidney, and lung tissues were fixed in 10% formalin (Sigma-Aldrich) for 48 h at 4°C and were then embedded in paraffin, using a routine histologic procedure, following infection. H&E staining was carried out according to the routine staining procedure (80).

### Induction PA14 antibiotic resistance assays.

PA14 was cultured in LB containing 0.25 × (25 μg/mL) antibiotics. Bacteria were centrifuged and increased by 25 μg/mL antibiotics with fresh LB every 8 h until the concentration of antibiotic was 1× (100 μg/mL). Then, 100 μg/mL antibiotics with fresh LB were continued to be added every 8 h to induce PA14 antibiotic resistance. PA14 antibiotic resistance was determined by detecting OD_600_ at 4 h intervals up to 96 h at 37°C with constant shaking.

### Statistical analysis.

All experiments were repeated three times unless stated otherwise. Data were presented as mean ± standard error of the mean (SEM). Statistical analysis was performed with GraphPad (GraphPad Software, La Jolla, CA), using a one-way analysis of variance (ANOVA) plus Tukey’s *post hoc* test. Statistically significant differences are indicated by *, *P* < 0.05.
