# Supplementary material for: Engineered Bacteriophages Containing Anti-CRISPR Suppress Infection of Antibiotic-Resistant P. aeruginosa
Source: Microbiol Spectr. 2022 Aug 16;10(5):e01602-22. doi: 10.1128/spectrum.01602-22 (PMC9602763; doi:10.1128/spectrum.01602-22)
Supplement: Supplemental file 1 — Supplemental material. Download spectrum.01602-22-s0001.pdf, PDF file, 1.0 MB [file spectrum.01602-22-s0001.pdf]

Figure S1

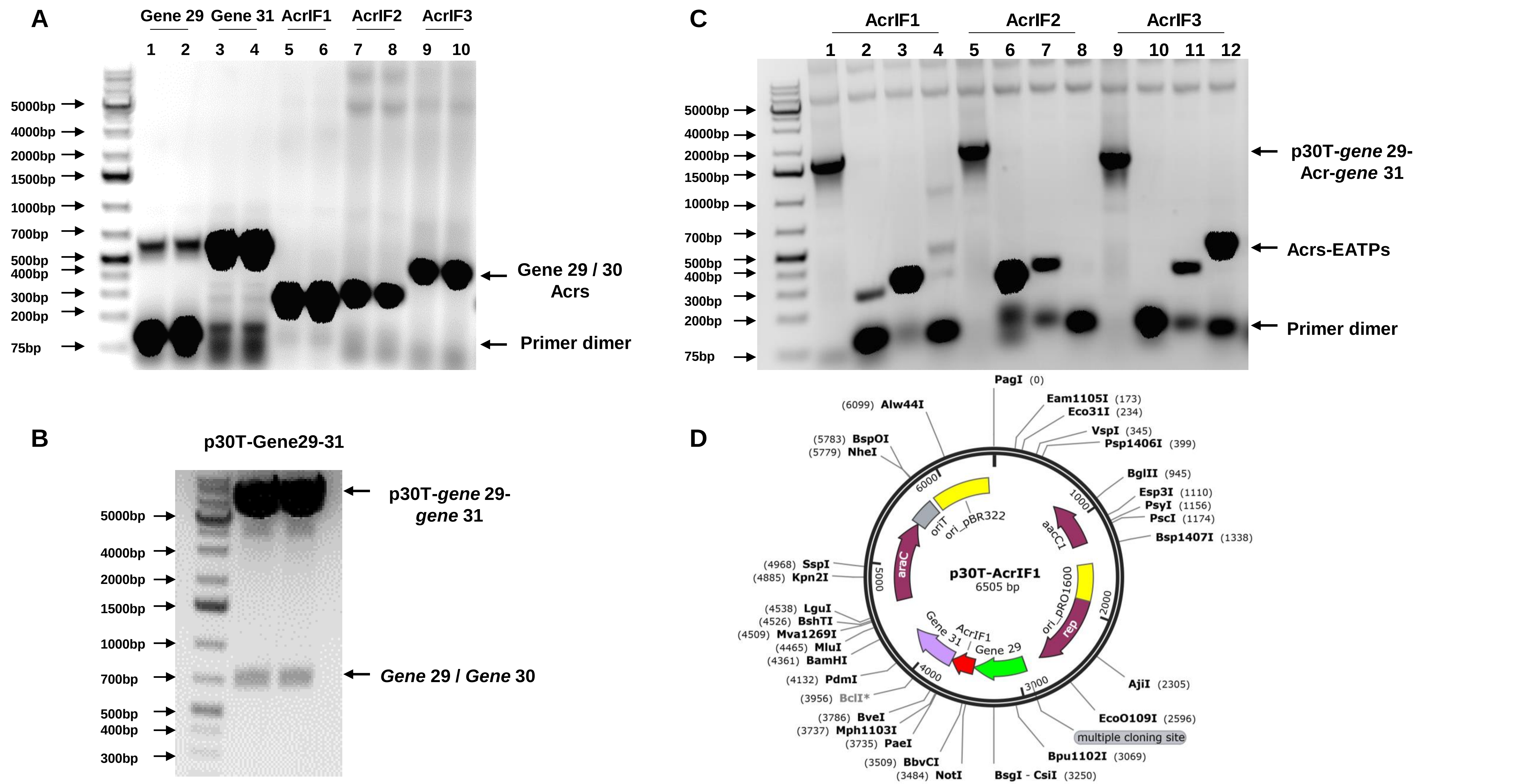

Figure S1. Engineered anti-CRISPR-containing phages (EATPs). The homology arms from the DMS3 (gene 30) genome up and downstream (Gene 29:578 bp, and Gene 31: 525 bp) and Acrs were amplified **(A)** and assembled into pHERD30T, was confirmed by enzyme digestion experiment (*PaeI* and NotI) **(B)**. **(C)** Recombined pHERD30T containing Acrs were transferred to PA14 and tested by PCR (1, 5, 9), several EATPs (2: DMS3$_{acrIF1}$; 6: DMS3$_{acrIF2}$, 11: DMS3$_{acrIF3}$; 3: DMS3m$_{acrIF1}$, 7: DMS3m$_{acrIF2}$, 12: DMS3m$_{acrIF3}$) were tested by PCR and agarose gel electrophoresis (4, 8, 10 is a negative control, which is the PCR product of PA14 without pHERD30T), and the sequences of the primers and anti-CRISPR genes used were listed in Table S2. **(D)** The plasmid of pHERD30T-Gene 29/30-AcrIF1 (p30T-AcrIF1) maps were created by SnapGene.

Figure S2

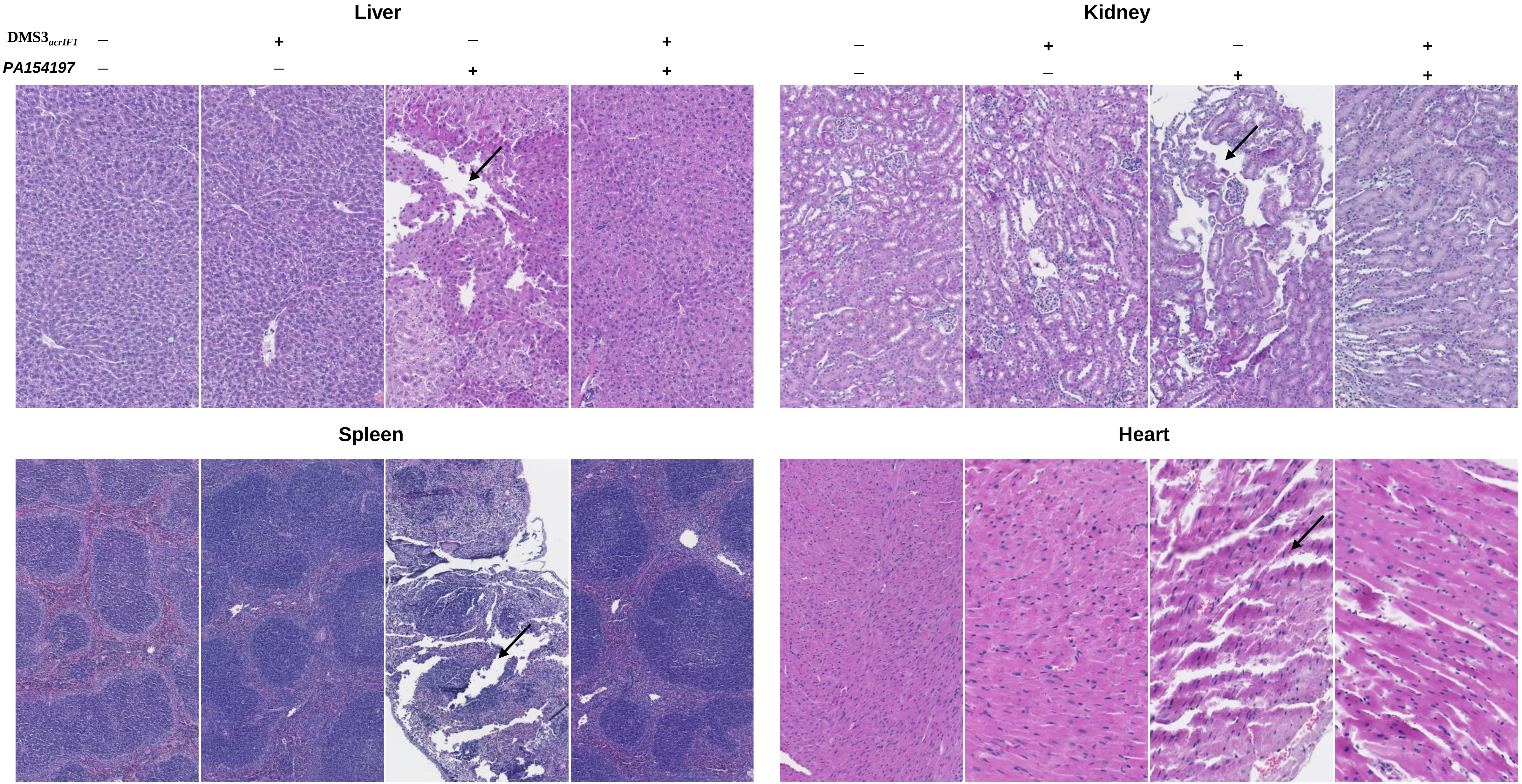

Figure S2. DMS3$_{macrIF1}$ significantly improve tissue damage in mice by H&E staining. C57BL/6N (n = 10) mice were anesthetized with ketamine (45 mg/kg) and intranasally instilled with 6×10^{6} CFU/25g clinically-isolated MDR *P. aeruginosa* PA154197 for 2 h, and intraperitoneally injected with 1.2×10^{6} PFU/mL DMS3$_{macrIF1}$ (MOI 0.2). The histological analysis was performed after completing the survival curve test at day 14. The cell rupture, inflammatory cell infiltration and tissue sclerosis were observed, and DMS3$_{macrIF1}$ treatment ameliorated tissues damage (magnified by 40 times) (Arrows represent typical tissue damage).

Figure S3

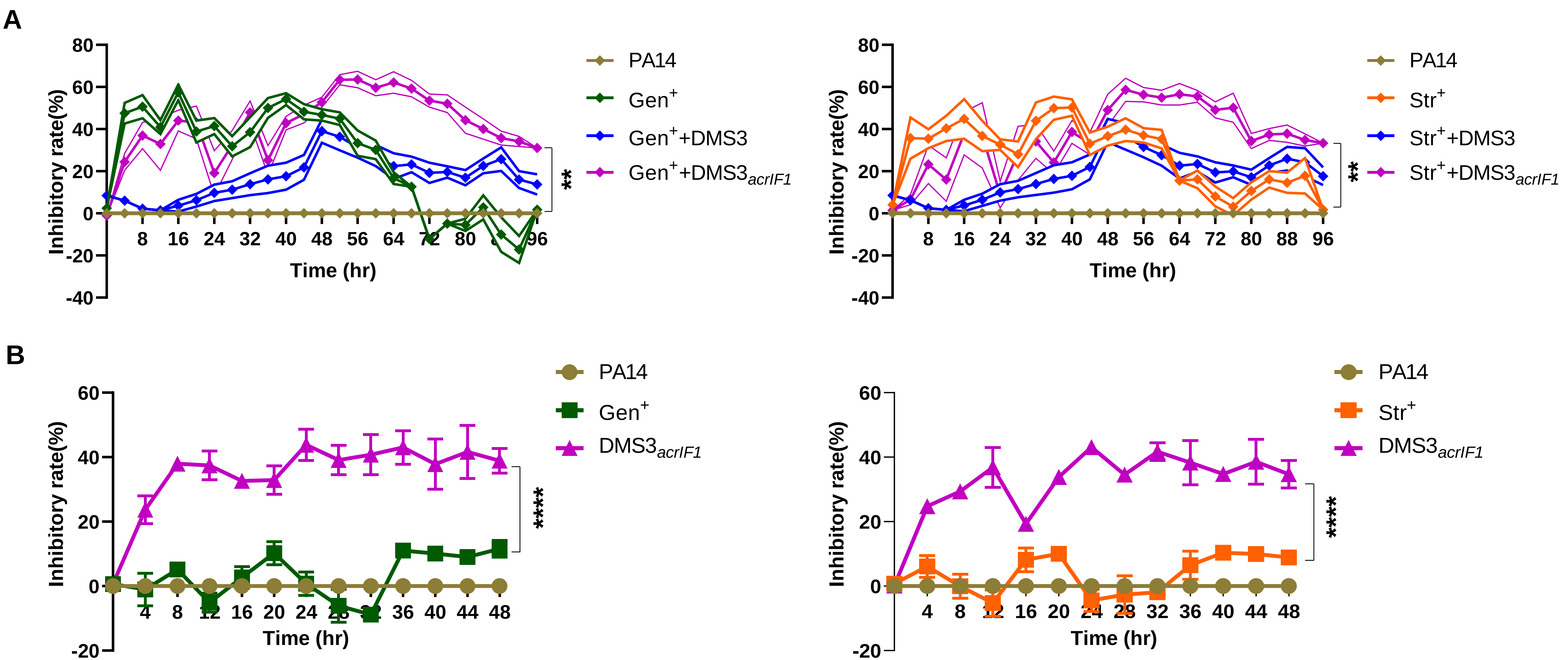

Figure S3. DMS3$_{macrIF1}$ contribute to suppressing *P. aeruginosa* antibiotic resistance. (A) PA14 (PA14: 5×10^{8} CFU/mL) was treated with gradient concentrations antibiotic (Gen^{+} or Str^{+}), gradient concentrations antibiotic and DMS3 (MOI 0.02) (Gen^{+}/Str^{+} + DMS3) or (Gen^{+}/Str^{+} + DMS3$_{macrIF1}$). The inhibitory rate (%) of DMS3$_{acrIF1}$ on PA14 were measured. **(B)** PA14 acquired antibiotic resistance was picked out and grown to the mid-logarithmic phase (OD 600 = 0.4-0.6) in lysogeny broth (LB) at 37°C with 220-rpm shaking. The inhibitory rate (%) of DMS3$_{acrIF1}$ on PA14 were measured after treatment with DMS3$_{macrIF1}$ (MOI 0.2) or 100 μg/mL Gen^{+} / Str^{+} antibiotics. Data were presented as mean ± standard error means (SEM) determined from biological triplicates, analyzed by one-way ANOVA compared with the control group (* $p<$ 0.05, ** $p<$0.01, ***$p<$0.001, **** $p<$0.0001).

Supplementary Table S1

No.	Origin	CRISPR/Cas system	Strain ID	Antibiotic resistance
1	Laboratory	I-F	PA14	Multidrug resistant
2	Clinically isolated	I-F	PA154197	Multidrug resistant
3	Clinically isolated	I-F	PA150567	--
5	Clinically isolated	I-F	PA150209	*oprD* gene disrupted
6	Clinically isolated	I-F&I-E	PA151345	Multiple plasmids
7	Clinically isolated	I-F&I-C	PA153837	--
8	Clinically isolated	I-F	PA130788	--
9	Clinically isolated	I-F	PA150577	- -
10	Clinically isolated	No	PA27853	--
11	Clinically isolated	No	PA152165	Multidrug resistant

Supplementary Table S2

Gene	Primer F (5'-3')	Primer R (5'-3')	Application
**1**	TAAGAAGGAGATATACATACGTGACAACTAGGATCG<br>ACGTC	GTTAGAGGCGTCAGGCTTCATGCATGCGCGGCGGC<br>CGCGTAAGGCTTCTATCCTTCTTGGCG	Amplification DMS3 Gene 29
**2**	CGCCAAGAAGGATAGAAGCCTTACGCGGCCGC<br>CGCGCATGC ATGAAGCCTGACGCCTCTAAC	GTAAAACGACGGCCAGTGCCCTTGAATCCAGGCGC<br>TGCCA	Amplification DMS3 Gene 31
**3**	TTACGCGGCCGC<br>ATGAAGTTCATCAAATACCTCAGCA	CGCGCATGC TCAGGGGTTTTCACGCCGGGAAATC	Amplification & test AcrIF1
**4**	TTACGCGGCCGC<br>ATGATCGCTCAGCAGCACAAAGATA	CGCGCATGC TTACTCCTCCTCGACCGATTCA	Amplification & test AcrIF2
**5**	TTACGCGGCCGC ATGAGCAACACGATTTCAGATCG	CGCGCATGC TCATGCCTGTTCCTCATACCG	Amplification & test AcrIF3
**6**	GCGTCACACTTTGCTATGCC	CGTTGTAAAACGACGGCCAG	Test Acrs in pHERK30T
**Gene 29**	GTGACAACTAGGATCGACGTCGAGCTGGACGACCAGGAGGTTCGCCAGCGCCTGGCGCTGCTGATGCGCTCGGTGACCGATACGCTGCCGGTCATGCGTGGCATCGCTG<br>CCGAGCTGCTCGCGGAAACTGAGTTCGCCTTTATGGACGAGGGGCCGGGCTGGCCGCAGCTCAGCCCCGTGACTGTCGCCGCTCGCGAGGCTAAGGGGCGTGGTCCAC<br>ACCCGATCCTCCAGGTCACAAACGCCCTGGCTCGCTCGGTCACGACCTGGGCGGATCGCAACGAGGCGGGAATCGGGTCCAACTTGGTCTATGCGGCCATCCACCAATTT<br>GGTGGCGACGCCGGCCGGGGTCACCAGGTCGAAATTCCTGCACGGCGGTATCTGCCTTTCGACGAAAACGGCCAACTGGCGGCCGGCGCTCGGCAGTCAATTCTGGAGA<br>TCGTCCTAACAGCCTTGAGCCGAAATCGGTAGTGGCCACTTTCGGACAAGCGGCACATTGTGCCTATTGCGAATTAGGCACAATGTGCCTAATCTAACGTCATGCCAGCCA<br>CAACGGCGAGGCGCCAAGAAGGATAGAAGCC		
**Gene 31**	ATGAAGCCTGACGCCTCTAACCACAACCCAGACCCGCGCTACCTGCGCGGGCTGCTCAAGAAAGCCGGCATCAGCCAGCGGCGCGCAGCCGAGCTGCTCGGCCTCAGTG<br>ACAGGGTGATGCGCTATTACCTGAGCGAGGACATCAAGGAGGGCTACCGCCCCGCGCCGTATACCGTCCAGTTCGCCCTGGAGTGCCTGGCGAACGACCCGCCATCTGC<br>GTGATCACCTGATCCGCCCGCAAACGCGCTACACGCGCCGAAACGGGGTTAGACGCTACCTCGCTCAGAGTCGGTGCGTTAACCCCGTTAGAGCCCCGTTAGAAATCGCT<br>CCAGCGCCATTCTTGTGCCAGGGTTTGGCCAGAAGATGGCGCCGGACGGTTTCCGCAGTCGTTGAACCCCTTCATGTGACCGCTGCTTTCTACCGTCGCCACCATTGGCG<br>GCATGGAAAAGAACCGCCTACTCGTTGCCATCGCCGCCTGCTCGTTCCAGCTTCCCCAGCTGGAGGATGGCAGCGCCTGGATTCAAG		
**AcrIF1**	AtgAAGTTCATCAAATACCTCAGCACCGCTCACCTGAACTATATGAATATCGCCGTTTACGAAAATGGCAGCAAAATCAAAGCCCGCGTTGAGAACGTCGTAAACGGCAAAAG<br>CGTTGGTGCTCGTGATTTTGACTCAACGGAGCAACTGGAATCCTGGTTTTATGGTCTGCCTGGCAGTGGCCTCGGTCGTATTGAAAACGCTATGAATGAGATTTCCCGGCGT<br>GAAAACCCCTGA		
**AcrIF2**	AtgATCGCTCAGCAGCACAAAGATACTGTCGCAGCGTGCGAAGCCGCCGAAGCCATCGCTATCGCTAAAGATCAGGTATGGGATGGCGAGGGCTATACCAAGTACACGTTC<br>GACGACAACAGCGTCCTGATCCAGTCGGGCACTACTCAGTATGCGATGGATGCCGACGACGCAGACAGCATCAAAGGCTATGCGGACTGGCTGGACGATGAGGCTCGCTC<br>CGCCGAAGCGTCGGAGATCGAGCGCCTGCTTGAATCGGTCGAGGAGGAGTAA		
**AcrIF3**	AtgAGCAACACCATTAGCGATCGTATTGTGGCGCGTAGCGTGATTGAAGCGGCGCGTTTTATTCAGAGCTGGGAAGATGCGGATCCGGATAGCCTGACCGAAGATCAGGTG<br>CTGGCGGCGGCGGGCTTTGCGGCGCGTCTGCATGAAGGCCTGCAGGCGACCGTGCTGCAGCGTCTGGTGGATGAAAGCAACCATGAAGAATATCGTGAATTTAAAGCGT<br>GGGAAGAAGCGCTGCTGAACGCGGATGGCCGTGTGGCGAGCAGCCCGTTTGCGGATTGGGGCTGGTGGTATCGTATTGCGAACGTGatgCTGGCGACCGCGAGCCAGAAC<br>GTGGGCGTGACCTGGGGCAGCCGTGTGCATGGCCGTCTGatgGCGATTTTTCAGGATAAATTTAAACAGCGTTATGAAGAACAGGCG		
